# Supplementary figures and images for: Assessing the impact of the Gamma variant on COVID-19 patient admissions in a southern Brazilian tertiary hospital—A comparison of dual pandemic phases
Source: PLoS One. 2023 Dec 8;18(12):e0291701. doi: 10.1371/journal.pone.0291701 (PMC10707562; doi:10.1371/journal.pone.0291701)

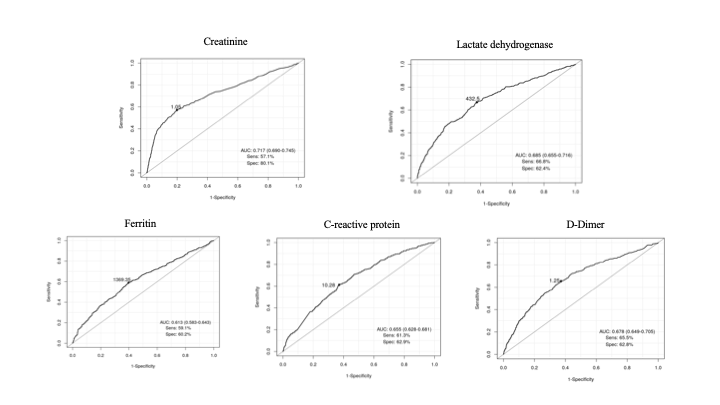

Supplement: S1 Fig — Note: ROC, receiver operating characteristic; AUC, area under the ROC curve; CRP, C-reactive protein. (TIF) [file pone.0291701.s001.tif]
