# Supplementary material for: Assessing the impact of the Gamma variant on COVID-19 patient admissions in a southern Brazilian tertiary hospital—A comparison of dual pandemic phases
Source: PLoS One. 2023 Dec 8;18(12):e0291701. doi: 10.1371/journal.pone.0291701 (PMC10707562; doi:10.1371/journal.pone.0291701)
Supplement: Attachment — Submitted filename: Response to Reviewers.docx [file pone.0291701.s003.docx]

October 31st, 2023

Dear Editor,

We hope this letter finds you well. We appreciate the time and effort you and the reviewers dedicated to evaluating our manuscript named **Assessing the impact of the Gamma variant on COVID-19 patient admissions in a Southern Brazilian tertiary hospital – A comparison of dual pandemic phases.** As requested, I would like to address the points raised in your feedback.

The manuscript has been carefully revised to address the major concerns of the reviewers, and we are now re-submitting it to PLOS ONE's. Our detailed point-by-point response and changes in the manuscript (Track changes) are provided below, and the online submission form will be updated accordingly.

If further adjustments are necessary, please do not hesitate to let us know, and we will promptly address them.

We appreciate your consideration and look forward to hearing from you.

Best Regards,

*Sonia Mara Raboni*

*Corresponding Author*

**Editor #:**

1. Please ensure that your manuscript meets PLOS ONE's style requirements, including those for file naming.

*The manuscript has been reviewed and adjusted to meet PLOS ONE's style requirements.*

2.Thank you for stating the following financial disclosure:
 "No - The funders had no role in study design, data collection and analysis, decision to publish, or preparation of the manuscript."

*The authors received no specific funding for this work. The research was conducted without any external financial support, and the authors were solely responsible for the study design, data collection, analysis, decision to publish, and manuscript preparation. As requested, the amended statements was included in the cover letter.*

3. Upon re-submitting your revised manuscript, please upload your study’s minimal underlying data set as either Supporting Information files or to a stable, public repository and include the relevant URLs, DOIs, or accession numbers within your revised cover letter.

*As requested, an anonymized spreadsheet containing the demographic, clinical, and laboratory data of research participants will be made available as Supporting Information file.*

4. We note that you have stated that you will provide repository information for your data at acceptance. Should your manuscript be accepted for publication, we will hold it until you provide the relevant accession numbers or DOIs necessary to access your data. If you wish to make changes to your Data Availability statement, please describe these changes in your cover letter and we will update your Data Availability statement to reflect the information you provide.

*Please check the answer to query 3.*

5. Please ensure that you include a title page within your main document. You should list all authors and all affiliations as per our author instructions and clearly indicate the corresponding author.

*A title page has been included into the main document, listing all authors and affiliations and indicating the corresponding author.*

6. Please upload a copy of Figure 1, to which you refer in your text on page 6

*A copy of Figure 1 was uploaded, which is referenced in the text on page 8 of the updated manuscript. The figure is now included as part of the submission.*

7. Please review your reference list to ensure that it is complete and correct. If you have cited papers that have been retracted, please include the rationale for doing so in the manuscript text, or remove these references and replace them with relevant current references. Any changes to the reference list should be mentioned in the rebuttal letter that accompanies your revised manuscript. If you need to cite a retracted article, indicate the article’s retracted status in the References list and also include a citation and full reference for the retraction notice.

*The reference list has been thoroughly examined and updated. None of the references was retracted.*

8. While revising your submission, please upload your figure files to the Preflight Analysis and Conversion Engine (PACE) digital diagnostic tool.

*The figure files have been uploaded to the Preflight Analysis and Conversion Engine (PACE) digital diagnostic tool to meet PLOS One figure specifications. The figures are provided in TIFF format, with a resolution of 300 PPI, and each file does not exceed 10MB in size, as per the requested requirements.*

**Reviewer #1:**

1) It will be nice, if the authors can have access to the vaccination status of these patients including the vaccine types and how many shots and compare these with the patient outcome. The paper is really valuable and acceptable for publication.

*The vaccination status of patients was not analyzed in this study due to the presence of data from periods without vaccination and extended periods with only one vaccine dose. Exploring the true impact of the vaccine in this situation proved challenging. Another study conducted at our institution specifically aims to address this analysis.*

**Reviewer #2:**

ABSTRACT: In line 8, it is not clear what CHC-UFPR is.

*In line 8 (39-40 in the updated document), the sentence was rewritten to maintain the number of words requested by the journal. The abbreviation CHC-UFPR has been expanded to its full form in M&M section.*

BACKGROUND: Lines 49-50 should be referenced.

In the updated document, the referenced lines 49-50 have been renumbered to 83-84. These lines have been appropriately referenced (7-9).

RESULTS: What are these higher inflammatory test values?

*The term "inflammatory tests" encompasses several nonspecific markers employed to evaluate the intensity of the inflammatory process. The markers evaluated were lactate dehydrogenase, ferritin, C-reactive protein, procalcitonin, and D-dimer, which are introduced in line 253-255 of this manuscript. Of note, the specific inflammatory tests under analysis are concisely outlined in Table 3, immediately after the sentence above.*

DISCUSSION: Line 292 would better read--patients ages.

*Line 292 (301-303 in the updated document) has been revised as suggested for improved clarity.*

**Reviewer #3:**

I have not concerns concerning the manuscript. I like the fact that datas gotten from the Research project are available. Though I would have appreciated that the Research is extended beyond Brazil.

*Acknowledging the reviewer's point, the pandemic significantly influenced healthcare services in our country, with Brazil experiencing one of the highest mortality rates. In our hospital, we established a distinctive protocol for consistent data collection and laboratory tests, even during the most critical periods. This protocol enabled the execution of the survey detailed in this manuscript. Regrettably, this approach was not universally adopted by other healthcare facilities, posing challenges for extending our findings beyond Brazil.*
